# Time course of serum uric acid accumulation and the risk of diabetes mellitus

**DOI:** 10.1038/s41387-021-00179-8

**Published:** 2022-01-10

**Authors:** Xue Tian, Anxin Wang, Yingting Zuo, Shuohua Chen, Licheng Zhang, Yuhan Zhao, Lulu Liu, Shouling Wu, Yanxia Luo, Jingli Gao

**Affiliations:** 1 Department of Epidemiology and Health Statistics, School of Public Health, Capital Medical University, Beijing, China; 2 Beijing Municipal Key Laboratory of Clinical Epidemiology, Beijing, China; 3 Department of Neurology, Beijing Tiantan Hospital, Capital Medical University, Beijing, China; 4 Department of Cardiology, Kailuan Hospital, North China University of Science and Technology, Tangshan, China; 5 China National Clinical Research Center for Neurological Diseases, Beijing Tiantan Hospital, Capital Medical University, Beijing, China; 6 Department of Intensive Medicine, Kailuan Hospital, North China University of Science and Technology, Tangshan, China

**Keywords:** Type 2 diabetes, Type 2 diabetes

## Abstract

**Background:**

The impact of long-term serum uric acid (SUA) exposure and time course of SUA accumulation on diabetes mellitus (DM) is unknown. This study aimed to evaluate the association of cumulative SUA (cumSUA) exposure and its accumulation time course with risk of DM.

**Methods:**

This prospective study included 46,434 participants without DM and underwent three examinations at 2006, 2008, and 2010. CumSUA from 2006 to 2010 was calculated, multiplying mean values between consecutive examinations by time intervals between visits. Time course of SUA accumulation was categorized as the slope of SUA versus time from 2006 to 2010, or by splitting the overall accumulation into an early (cumSUA_06-08_) and late accumulation (cumSUA_08-10_).

**Results:**

During 6.99 years of follow-up, we identified 2971 incident DM cases. In the fully adjusted model, a higher risk of DM was observed in participants with the highest quartile of cumSUA (hazard ratio [HR], 1.31; 95% confidence interval [CI], 1.17–1.46), cumulative burden >0 (HR, 1.23; 95% CI, 1.08–1.40), and with 6 year of hyperuricemia exposure duration (HR, 1.25; 95% CI, 1.01–1.55). When considering the time course of SUA accumulation, participants with a negative slope (HR, 1.05; 95% CI, 1.01–1.12), or combined with cumSUA ≥ median and a negative slope had elevated risk of DM (HR, 1.58; 95% CI, 1.18–2.11).

**Conclusions:**

Incident DM risk depends on cumulative exposure of SUA and time course of SUA accumulation. Early SUA accumulation resulted in a greater risk increase compared with later accumulation, emphasizing the importance of optimal SUA control early in life.

## Introduction

Diabetes mellitus (DM) is a worldwide public health problem [1]. In China, the prevalence of total DM was 12.8% and the healthcare cost associated with diabetes is 110 billion international dollars (purchasing power parity) in 2017 [2]. DM is also a well-established risk factor for cardiovascular disease, renal dysfunction, dementia, and all-cause mortality [3–5]. Primary prevention strategies including early detection and control risk factors are necessary to reduce the impeding incidence of DM and its related complications.

Serum uric acid (SUA), the end product of purine metabolism by xanthine oxidoreductase, is correlated with many recognized cardiovascular risk factors. Animal experiments [6] and few intervention studies [7] in human have shown that reducing SUA might improve insulin resistance, which raises great interest in the relation between SUA and DM. In recent decades, a number of prospective observational studies reported a positive association between SUA levels and incident DM risk [8–11]. In contrast, some indicated a negative association [12] or supported bystander role of SUA in the development of DM [13–15]. The discrepancy may be attributable to that the majority of these published studies only analyzed the single measurement of SUA levels, failing to take the potential effect of long-term cumulative exposure of SUA into account, which might bias the true relation between SUA and DM risk. Considering the methodological limitations, several recent studies have investigate the effect of change in hyperuricemia status on DM risk [15, 16], however, changes in SUA in the aforementioned study were defined on the basis of 2 measurements of SUA levels and were unable to account for the longitudinal patterns of SUA over a period. On this consideration, the method of cumulative metrics has been proposed, which can capture both the duration and intensity of SUA over several years [17–20]. Furthermore, incorporation both the SUA levels and exposure duration into a single risk parameter for future DM is intuitively appealing, although a data-based demonstration of the utility of this metric is insufficient. Additionally, it is also unclear whether the time course of SUA accumulation plays an important role in modulating the risk conferred by a give overall exposure.

Therefore, we conducted the present study to (1) quantify the association of cumulative exposure to SUA (cumSUA) with DM risk; (2) evaluate the impact of cumulative burden of SUA; (3) hyperuricemia exposure duration; (4) and the modulated effect of time course of SUA accumulation on DM risk based on a large community-based prospective cohort study.

## Subjects and methods

### Study design and population

The Kailuan study is an ongoing prospective cohort study conducted in Tangshan City, China. The study was designed to investigate the risk factors for common noncommunicable diseases and the detailed study design and characteristics of the study population have been described previously [21, 22]. In brief, a total of 101,510 participants (aged 18–98 years, 81,110 men and 20,400 women) completed the first survey from June 2006 to October 2007 and underwent a comprehensive biennial health examination thereafter. In the current, we calculated cumSUA using SUA levels in 2006, 2008, and 2010 to predict incident DM after 2010, the time line of the study is presented in Fig. S1. We excluded 44,677 participants who did not have three times health examinations, 8693 participants who were diagnosed with DM (the criteria for DM was described below) to minimize the possible effect of reverse causality, and 1706 participants with missing data on SUA. Consequently, 46,434 participants were included in the present analysis (Fig. S2). A comparison of included and excluded participant characteristics owing to missing data or follow-up is presented in Table S1. The study was performed according to the guidelines of the Helsinki Declaration and was approved by the Ethics Committee of Kailuan General Hospital and Beijing Tiantan Hospital. All participants provided written informed consent.

### Calculation of cumSUA, cumulative burden, duration of hyperuricemia exposure, and time course of SUA accumulation

Blood samples were collected following an 8–12 h overnight fast at baseline and was then measured biennially. SUA concentrations were measured at the central laboratory in the Kailuan General Hospital. The inter-assay coefficient of variation for each measurement conducted using an auto analyzer (Hitachi 7600, Tokyo, Japan) with an oxidase method was ≤6.0%.

**Fig. 1:**
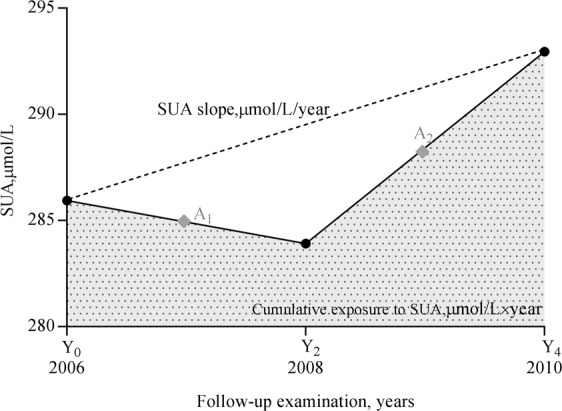
Cumulative SUA and SUA slope calculated across 3 examinations in 1 participants. Average SUA between consecutive examinations as A1 and A2. Cumulative SUA was calculated as (A1 × time06–08 + A2 × time08–10), showed by the dotted area, μmol/L × year. SUA slope was obtained using a linear regression, SUA values at follow-up visits were used to calculate the SUA. SUA serum uric acid.

CumSUA was defined as the sum of products of average SUA in 2 consecutive follow-up examinations multiplied by the follow-up interval in years [17–19], detailed in Fig. 1, and the formula calculated as follows:

$${{{\mathrm{CumSUA}}}} = [({{{\mathrm{SUA}}}}_{2006} + {{{\mathrm{SUA}}}}_{2008})/2 \times {{{\mathrm{time}}}}_{2006 - 2008}] + [({{{\mathrm{SUA}}}}_{2008} + {{{\mathrm{SUA}}}}_{2010})/2 \times {{{\mathrm{time}}}}_{2008 - 2010}]$$

where SUA_2006_, SUA_2008_, and SUA_2010_ indicate SUA at the first (baseline), second, and third examinations, and time_2006–2008_ and time_2008–2010_ indicate the participant-specific time intervals between consecutive examinations in years [23]. The means of time_2006–2008_ and time_2008–2010_ were 2.10 and 1.95 years, respectively.

Cumulative burden is defined as the weighted sum of the portion of 2 adjacent, average measurements that falls above the cutoff value that is then multiplied by time intervals between consecutive examinations in years:

$${{{\mathrm{Cumulative burden}}}} = [({{{\mathrm{SUA}}}}_{2006} + {{{\mathrm{SUA}}}}_{2008})/2 - {{{\mathrm{cutoff}}}}] \times {{{\mathrm{time}}}}_{2006 - 2008} + [({{{\mathrm{SUA}}}}_{2008} + {{{\mathrm{SUA}}}}_{2010})/2 - {{{\mathrm{cutoff}}}}] \times {{{\mathrm{time}}}}_{2008 - 2010}$$

Hyperuricemia was defined as a SUA level ≥420 μmol/L for men and ≥360 μmol/L for women [24], which was the cutoff in the formula. If the values of the cumulative burdens between 2 consecutive examinations were less than 0 this value would be considered as 0 [25].

Hyperuricemia exposure duration was defined as the times of visits with hyperuricemia at visit 2006, 2008, and 2010. We give 1 point to hyperuricemia, 0 point for normouricemia at each visit, therefore, hyperuricemia exposure duration was ranged from 0 years (never had hyperuricemia), 2 years (had hyperuricemia once), 4 years (had hyperuricemia twice), and 6 years (had hyperuricemia at all three study visits) [26].

Time course of SUA accumulation was categorized in 2 ways: a slope of SUA versus time from 2006 to 2010 using the linear regression and the least-squares principle, with a positive or negative slope indicating an increase or decrease in SUA over time (illustrated in Fig. 1); or alternatively, the cumSUA between 2006 to 2008 (cumSUA06-08), and 2008–2010 (cumSUA08-10) were calculated as early and late SUA exposure measures, respectively [27].

### Follow-up and assessment of DM

For each participant, follow-up started from 2010 and ended at the occurrence of DM, death, or the end of the follow-up on 31 December 2017, which event came first. DM was defined as either FBG ≥ 7.0 mmol/L, self-reported of a physician diagnosis, or self-reported use of antidiabetic medication.

### Assessment of covariates

All individuals completed a standardized questionnaire that collected information on their demographic characteristics, lifestyle habits, and medical history. Active physical activity was defined as exercising more than four times a week and at least 20 min each time. Body mass index (BMI) was calculated by dividing weight (kilogram) by squared height (meter^2^). Blood pressure were measured two times with the participants in the seated position with a mercury sphygmomanometer according to the JCN7 recommendation. The average of two readings was used for the analyses.

The fasting blood samples were tested using a Hitachi 747 auto-analyzer (Hitachi, Tokyo, Japan) at the central laboratory of the Kailuan Hospital, including fasting blood glucose (FBG), serum lipid profiles, serum creatinine, and high-sensitivity C-reactive protein (hs-CRP). Estimated glomerular filtration rate (eGFR) was calculated using the creatinine-based Chronic Kidney Disease Epidemiological Collaboration equation [28].

Hypertension was defined as any self-reported hypertension or use of antihypertensive drug, or blood pressure ≥140/90 mmHg. Dyslipidemia was defined as any self-reported history or use of lipid-lowering drugs, or serum total cholesterol ≥5.17 mmol/L or triglyceride ≥1.69 mmol/L or low-density lipoprotein cholesterol ≥3.62 mmol/L or high-density lipoprotein cholesterol ≤1.04 mmol/L.

### Statistical analysis

Continuous variables were described as mean ± standard deviation and differences across baseline groups were compared using Student’s *t*-test or ANOVA. Categorical variables were described as frequency with percentage and were compared using Chi-square test. Person-years was determined from the date when the message was collected to either the date of incident DM, death, or the date of participating in the last examination in the analysis, whichever came first.

Cox proportional hazard regression was applied to calculate he hazard ratio (HR) and 95% confidence interval (CI) for the risk of DM. According to Schoenfeld residuals and log-log inspection, our models met the proportional assumption criteria. Four models were constructed systematically, model 1 was unadjusted; model 2 was adjusted for age and sex; model 3 was further adjusted for education, income, smoking status, drinking status, history of hypertension and dyslipidemia, BMI, systolic blood pressure, diastolic blood pressure, and FBG; model 4 was further adjusted for antihypertensive agents, diuretics, lipid-lowering agents, eGFR, and hs-CRP. For each model, a trend test was performed after the median value of each quartile was entered into the model and treated as a continuous variable. The restricted cubic spline models were with 4 knots (at the 5th, 35th, 65th, and 95th percentiles) were conducted to test whether there was a dose-response or nonlinear association of cumSUA as a continuous variable with the risk of DM, adjusting for all the covariates above-mentioned. When considering the combined effect of SUA accumulation and its time course, participants were divided into 4 categories according to the median of cumSUA (1118.7 μmol/L × year) and the direction of slope (negative or positive). The association between combined effect of SUA accumulation and its time course and DM was also assessed using multivariable Cox proportional hazard models.

To test the robustness of our findings, we further conducted several sensitivity analyses. First, competing risk model was applied considering non-DM deaths as competing risk events. Second, to explore the potential impact of reverse causality, we excluded the outcome events that occurred within the first 2 years of the follow-up period. Third, considering diuretics may have a potential effect on SUA concentration, we excluded participants who used diuretics during the whole study period.

All analyses were conducted using SAS version 9.4 (SAS Institute Inc., Cary, NC, USA). A two-sided *P* < 0.05 was considered statistically significant.

## Results

### Baseline characteristics

The mean age of the enrolled participants was 48.66 ± 11.97 years, 75.75% were men.

Baseline characteristics according to the combined effect of cumSUA accumulation and its time course are presented in Table 1. Compared with participants with cumSUA < median, slope ≥ 0, participants in other groups were more likely to be older, men, educated, had higher income, more current smokers, current alcohol users, active physical activity, a higher prevalence of hypertension, dyslipidemia, more likely to take antihypertensive agents, lipid-lowering agents, a higher level of BMI, FBG, SBP, DBP, hs-CRP, and a lower level of eGFR. The characteristics of participants according to quartiles of cumSUA are presented in Table S2. There was also a significant difference among the four categories in all the baseline characteristics.

**Table 1:** Baseline characteristics of participants stratified by cumSUA and SUA slope.

Characteristics	Overall	CumSUA < median, slope ≥ 0	CumSUA < median, slope < 0	CumSUA ≥ median, slope ≥ 0	CumSUA ≥ median, slope < 0	*P*-value
No. of participants (%)	46,434	12,199 (26.27)	11,017 (23.73)	13,497 (29.07)	9721 (20.94)	
Age, years	48.66 ± 11.97	46.83 ± 10.95	46.23 ± 11.07	49.16 ± 12.49	53.03 ± 12.18	<0.0001
Male, *n* (%)	35,175 (75.75)	7428 (60.89)	7685 (69.76)	11,786 (87.32)	8276 (85.14)	<0.0001
High school or above, *n* (%)	11,049 (24.33)	2670 (21.89)	2289 (20.78)	3604 (26.70)	2486 (25.57)	<0.0001
Income ≥ 800RMB, *n* (%)	7125 (15.70)	1410 (11.56)	1312 (11.91)	2478 (18.36)	1925 (19.80)	<0.0001
Current smoker, *n* (%)	15,829 (34.88)	3009 (24.67)	3245 (29.45)	5643 (41.81)	3932 (40.45)	<0.0001
Current alcohol, *n* (%)	18,126 (39.92)	3379 (27.70)	3521 (31.96)	6580 (48.75)	4646 (47.79)	<0.0001
Active physical activity, *n* (%)	41,340 (91.23)	10,772 (88.30)	9890 (89.77)	11,863 (87.89)	8815 (90.68)	<0.0001
Hypertension, *n* (%)	4652 (10.02)	651 (5.34)	605 (5.49)	1747 (12.94)	1649 (16.96)	<0.0001
Dyslipidemia, *n* (%)	2526 (5.44)	375 (3.07)	289 (2.62)	951 (7.05)	911 (9.37)	<0.0001
Antihypertensive agents, *n* (%)	4069 (8.76)	540 (4.43)	495 (4.49)	1556 (11.53)	1478 (15.20)	<0.0001
Lipid-lowering agents, n (%)	367 (0.79)	48 (0.39)	37 (0.34)	147 (1.09)	135 (1.39)	<0.0001
Body mass index, kg/m^2^	24.86 ± 3.42	24.26 ± 3.36	24.44 ± 3.38	25.36 ± 3.34	25.39 ± 3.41	<0.0001
FBG, mmol/L	5.00 ± 0.66	4.97 ± 0.65	5.06 ± 0.67	5.01 ± 0.65	4.98 ± 0.66	<0.0001
SBP, mmHg	127.42 ± 19.63	124.71 ± 18.87	125.83 ± 18.56	129.09 ± 20.27	130.38 ± 20.04	<0.0001
DBP, mmHg	82.17 ± 11.28	80.85 ± 11.03	81.68 ± 10.95	82.87 ± 11.53	83.44 ± 11.28	<0.0001
eGFR, mL/min/1.73 m^2^	84.38 ± 24.52	85.77 ± 24.54	85.47 ± 27.59	84.44 ± 23.64	81.29 ± 21.48	<0.0001
hs-CRP, mg/L	2.25 ± 6.19	2.19 ± 7.20	1.58 ± 3.99	2.75 ± 5.90	2.39 ± 7.02	<0.0001

*CumSUA* cumulative serum uric acid, *DBP* diastolic blood pressure, *eGFR* estimated glomerular filtration rate, *FBG* fasting blood glucose, *hs-CRP* high-sensitivity C-reactive protein, *SBP* systolic blood pressure, *SUA* serum uric acid.

### Association of cumSUA, cumulative burden, and hyperuricemia exposure duration with the risk of DM

**Fig. 2:**
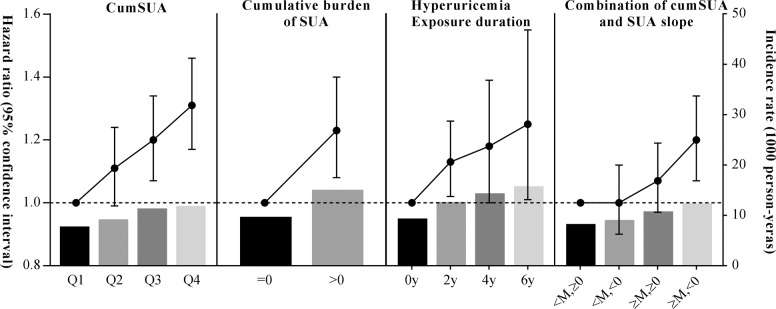
Incidence rate and hazard ratio of diabetes mellitus according to cumulative SUA indexes. cumSUA, cumulative serum uric acid, SUA serum uric acid. The four categories of combination of cumSUA and SUA slope were as follows: <M, ≥0: cumSUA < Median, and slope ≥ 0; <M, <0: cumSUA < Median, and slope < 0; ≥M, ≥0: cumSUA ≥ Median, and slope ≥ 0; ≥M, <0: cumSUA ≥ Median, and slope < 0. Adjusted for age, sex, education, income, smoking status, drinking status, history of hypertension and dyslipidemia, antihypertensive agents, hypoglycemic agents, diuretics, lipid-lowering agents, body mass index, fasting blood glucose, systolic blood pressure, diastolic blood pressure, estimated glomerular filtration rate, and high-sensitivity C-reactive protein.

**Fig. 3:**
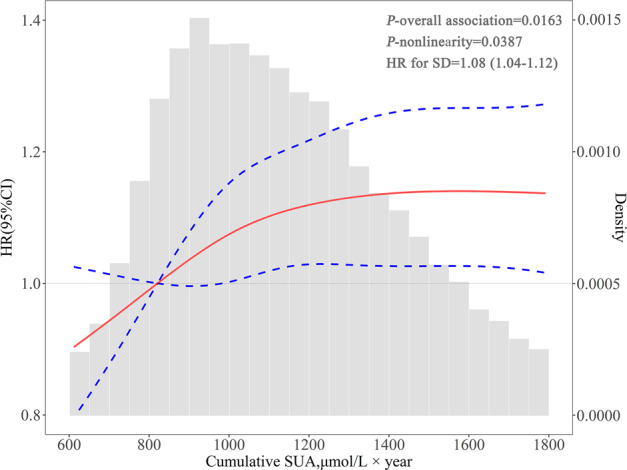
Cumulative SUA on a continuous scale and risk of diabetes mellitus. Hazard ratio (solid line) and 95% confidence interval (dash lines) from Cox regression using restricted cubic spline regression with 4 knots with placed at the 5th, 35th, 65th, 95th percentiles. Multivariate adjustment was for age, sex, education, income, smoking status, drinking status, history of hypertension and dyslipidemia, antihypertensive agents, hypoglycemic agents, diuretics, lipid-lowering agents, body mass index, fasting blood glucose, systolic blood pressure, diastolic blood pressure, estimated glomerular filtration rate, and high-sensitivity C-reactive protein.

During a median follow-up of 6.99 years, 2971 (6.40%) incident DM were detected. The associations of cumulative SUA parameters with the risk of DM are presented in Table 2 and Fig. 2. The incidence rate per 1000 person-years of DM increased across the cumSUA quartiles, ranging from 7.41 (95% CI, 6.83–8.03) in the lowest quartile to 11.50 (95% CI, 10.70–12.30) in the highest quartile group. The association remained significant even after adjustment for potential variables, the fully adjusted HR (Model 4) was 1.31 (95% CI, 1.17–1.46; *P* for trend < 0.0001) in the lowest quartile versus the highest quartile of cumSUA. Multivariable-adjusted spline regression models yielded the same pattern of results when cumSUA was treated as a continuous variable (HR for per 1 SD was 1.08 [95% CI, 1.04–1.12]; Fig. 3).

**Table 2:** Hazards ratios and 95% confidence interval for the risk of diabetes mellitus stratified by cumulative SUA indexes.

Index	Case, *n* (%)	Incidence rate^a^	Model 1	Model 2	Model 3	Model 4
Cumulative SUA, μmol/L × year						
Q1	583 (5.02)	7.41 (6.83–8.03)	Reference	Reference	Reference	Reference
Q2	686 (5.91)	8.82 (8.19–9.51)	1.18 (1.06–1.32)	1.14 (1.02–1.27)	1.12 (1.00–1.25)	1.11 (0.99–1.24)
Q3	836 (7.20)	11.00 (10.30–11.80)	1.48 (1.33–1.64)	1.37 (1.23–1.53)	1.22 (1.09–1.37)	1.20 (1.07–1.34)
Q4	866 (7.46)	11.50 (10.70–12.30)	1.53 (1.38–1.70)	1.42 (1.27–1.59)	1.32 (1.18–1.47)	1.31 (1.17–1.46)
*P*_trend_			<0.0001	<0.0001	<0.0001	<0.0001
Cumulative burden of SUA						
=0	2714 (6.21)	9.34 (9.00–9.70)	Reference	Reference	Reference	Reference
>0	257 (9.39)	14.70 (13.00–16.70)	1.56 (1.37–1.77)	1.49 (1.31–1.70)	1.26 (1.10–1.43)	1.23 (1.08–1.40)
*P*-value			<0.0001	<0.0001	0.0006	0.0022
Exposure duration of hyperuricemia^b^						
0 year	2336 (6.01)	9.01 (8.65–9.38)	Reference	Reference	Reference	Reference
2 year	390 (7.95)	12.30 (11.10–13.50)	1.35 (1.21–1.50)	1.33 (1.19–1.48)	1.14 (1.02–1.27)	1.13 (1.02–1.26)
4 year	153 (8.95)	14.00 (12.00–16.40)	1.53 (1.30–1.81)	1.48 (1.25–1.74)	1.21 (1.02–1.42)	1.18 (1.00–1.39)
6 year	92 (9.83)	15.40 (12.60–18.90)	1.65 (1.34–2.04)	1.58 (1.28–1.95)	1.30 (1.05–1.61)	1.25 (1.01–1.55)
*P*_trend_			<0.0001	<0.0001	<0.0001	0.0014
Combination of cumulative SUA and SUA slope^c^						
CumSUA < median, slope ≥ 0	657 (5.39)	7.92 (7.34–8.55)	Reference	Reference	Reference	Reference
CumSUA < median, Slope < 0	655 (5.95)	8.73 (8.09–9.42)	1.10 (0.98–1.22)	1.10 (0.99–1.23)	1.01 (0.91–1.13)	1.00 (0.90–1.12)
CumSUA ≥ median, Slope ≥ 0	911 (6.75)	10.40 (9.74–11.10)	1.29 (1.17–1.43)	1.21 (1.09–1.35)	1.08 (0.97–1.20)	1.07 (0.97–1.19)
CumSUA ≥ median, Slope < 0	748 (7.69)	12.00 (11.20–12.90)	1.49 (1.34–1.65)	1.31 (1.18–1.46)	1.22 (1.09–1.36)	1.20 (1.07–1.34)
*P*_trend_			<0.0001	<0.0001	<0.0001	<0.0001

Model 1: unadjusted.

Model 2: adjusted for age and sex.

Model 3: further adjusted for education, income, smoking status, drinking status, history of hypertension, and dyslipidemia, body mass index, fasting blood glucose, systolic blood pressure, diastolic blood pressure.

Model 4: further adjusted for antihypertensive agents, diuretics, hypoglycemic agents, lipid-lowering agents, estimated glomerular filtration rate, and high-sensitivity C-reactive protein.

*SUA* serum uric acid, *cumSUA* cumulative serum uric acid.

^a^Incidence rate per 1000 person-years.

^b^Hyperuricemia was defined as serum uric acid > 420 μmol/L in men and >360 μmol/L in women.

^c^Median of cumulative SUA was 1118.70 μmol/L × year.

Similar results were observed for cumulative burden of SUA and hyperuricemia exposure duration. For those with cumulative burden >0 compared with cumulative burden ≤0, the risk of DM increased by 123% (HR, 1.23; 95% CI, 1.08–1.40, *P* = 0.0022). When comparing the participants with the longest to the shortest hyperuricemia exposure duration, participants with a 6 year exposure duration of hyperuricemia had 1.25-fold higher risk of MI (HR, 1.25; 95% CI, 1.01–1.55, *P* for trend = 0.0014). The sensitivity analyses with competing risk model, excluding outcome within the first year of follow-up (*n* = 858), or excluding participants who used diuretics at baseline and during the follow-up (*n* = 1455) did not materially change the results (Table 3).

**Table 3:** Sensitivity analyses for the association between cumulative SUA and diabetes mellitus.

Index	Sensitivity analysis^a^	Sensitivity analysis^b^	Sensitivity analysis^c^
Cumulative SUA, μmol/L × year			
Q1	Reference	Reference	Reference
Q2	1.13 (1.01–1.26)	1.08 (0.96–1.22)	1.14 (1.01–1.27)
Q3	1.21 (1.08–1.36)	1.13 (1.00–1.28)	1.23 (1.10–1.38)
Q4	1.33 (1.19–1.48)	1.22 (1.08–1.37)	1.33 (1.19–1.49)
Cumulative burden of SUA			
=0	Reference	Reference	Reference
>0	1.22 (1.06–1.39)	1.18 (1.03–1.37)	1.24 (1.08–1.42)
Exposure duration of hyperuricemia^d^			
0 year	Reference	Reference	Reference
2 year	1.14 (1.02–1.27)	1.13 (0.94–1.36)	1.14 (1.02–1.28)
4 year	1.18 (1.00–1.40)	1.16 (1.03–1.30)	1.24 (1.04–1.48)
6 year	1.26 (1.02–1.56)	1.27 (1.01–1.60)	1.28 (1.01–1.56)
Combination of cumulative SUA and SUA slope^e^			
CumSUA < median, slope ≥ 0	Reference	Reference	Reference
CumSUA < median, Slope < 0	1.00 (0.89–1.11)	1.12 (0.99–1.26)	1.00 (0.89–1.12)
CumSUA ≥ median, Slope ≥ 0	1.08 (0.98–1.21)	1.12 (1.00–1.26)	1.09 (0.98–1.22)
CumSUA ≥ median, Slope < 0	1.18 (1.06–1.32)	1.31 (1.16–1.47)	1.18 (1.05–1.32)

Adjusted for age, sex, education, income, smoking status, drinking status, history of hypertension and dyslipidemia, antihypertensive agents, hypoglycemic agents, diuretics, lipid-lowering agents, body mass index, fasting blood glucose, systolic blood pressure, diastolic blood pressure, estimated glomerular filtration rate, and high-sensitivity C-reactive protein.

*SUA* serum uric acid, *cumSUA* cumulative serum uric acid.

^a^Sensitivity analysis was performed using competing risk model considering death as a competing risk.

^b^Sensitivity analysis was performed by excluding outcome within the first year of follow-up (*n* = 858).

^c^Sensitivity analysis was performed by excluding participants who used diuretics at baseline and during the follow-up (*n* = 1455).

^d^Hyperuricemia was defined as serum uric acid >420 μmol/L in men and >360 μmol/L in women.

^e^Median of cumulative SUA was 1118.70 μmol/L × year.

### Time course of SUA accumulation and risk of DM

The associations of the time course of SUA accumulation evaluated by adding the slope of SUA time course over 2006–2010 to the analysis, or by splitting the overall accumulation into an early (cumSUA_06-08_) and late accumulation (cumSUA_08-10_) in the models are presented in Table 4. After adjusted for potential covariates, participants with negative slope of SUA time course (decreasing SUA trend) had elevated DM risk (HR, 1.05; 95% CI, 1.01–1.12; *P* = 0.0303), compared with participants with positive slope of SUA time course (increasing SUA trend). Consistently, the later accumulation was not associated with DM after adjusted for early accumulation in the multivariable model (*P* = 0.2161).

**Table 4:** Association of time course of SUA accumulation with risk of diabetes.

	HR (95% CI)	*P-*value
Slope of SUA^a^		
<0	1.05 (1.01–1.12)	0.0303
≥0	Reference	
CumSUA06-08	1.03 (1.01–1.05)	0.0012
CumSUA08-10^b^	1.00 (0.96–1.02)	0.2161

Adjusted for age, sex, education, income, smoking status, drinking status, history of hypertension and dyslipidemia, antihypertensive agents, diuretics, lipid-lowering agents, body mass index, systolic blood pressure, diastolic blood pressure, fasting blood glucose, estimated glomerular filtration rate, and high-sensitivity C-reactive protein.

*SUA* serum uric acid, *cumSUA06-08* cumulative serum uric acid between 2006 and 2008, *cumSUA08-10* cumulative serum uric acid between 2008 and 2010, *HR* hazard ratio, *CI* confidence interval.

^a^Further adjusted for cumSUA between 2006 and 2010.

^b^Further adjusted for cumSUA between 2006 and 2008.

When considering the combined effect of cumSUA accumulation and its time course, in the fully adjusted model, participants with cumSUA ≥ median and a negative slope of SUA time course had the highest risk of DM (HR, 1.20; 95% CI, 1.07-1.34), compared with those with cumSUA< median and a positive slope of SUA time course (Table 2). Sensitivity analysis showed the similar results (Table 3).

## Discussion

This study shows that the risk of future incident DM is associated with the total umulative exposure of SUA. Importantly, this risk is modulated by the time course of SUA accumulation. Notably, our data suggest that cumSUA accumulated early confers greater risk of DM than when the same cumSUA is accumulated later. These findings underscore the importance of optimal SUA early in life, because lower SUA later, even when low enough to result in the same accumulation at the same time point, does not fully reverse risk acquired earlier. However, it is important to recognize that these data do not in any way suggest that there is no primary prevention benefit to lowering SUA no matter when elevated SUA lowering is started. Our results indicate an apparent persistent increase in later DM risk conferred by high SUA levels experienced early in life.

The present analyses showed that participants with high cumSUA levels, or a higher cumulative burden of SUA over time had a higher risk of developing DM, relative to their counterparts with lower cumSUA over time. Most previous studies but not all, which were generally based on a single SUA assessment, generated consistent results regarding the association between SUA and DM risk. For example, data from the Framingham Heart Study original and offering cohorts showed that individuals with higher SUA are at a higher future risk of DM independent of other known risk factors [9]. The positive association was also supported by the Rotterdam Study [29], the National Health Interview Survey [10], the China Stroke Primary Prevention Trial [11], and some other cohort studies [30, 31]. Of note, a single measurement of SUA in these studies may not adequately reflect the longitudinal variation and cumulative burden associated with elevated SUA level. In contrast, the measurement of cumSUA could capture both the duration and intensity of SUA exposure over several decades. Our findings taken together with previous studies suggested that not only baseline SUA, but also a longitudinal cumSUA is important in predicting the risk of DM.

In addition, our study investigated the association between exposure duration of hyperuricemia and DM risk, the results showed that the risk of DM increased with hyperuricemia exposure duration. Hyperuricemia is a metabolic problem that has become increasing common worldwide and its association with DM was observed in meta-analysis, as well as in many epidemiological studies [32, 33]. However, the evidence on the effect of hyperuricemia duration on DM risk was scarce. Experimental studies revealed that hyperuricemia is associated with kidney damage via stimulating RAS activity and promoting endothelial damage along with oxidative stress [34], which are important contributors in the development of DM. Furthermore, hyperuricemia is a disease with stepwise progression [35], the damage in organisms is aggravating with the duration of hyperuricemia, thus leading to increasing DM risk with hyperuricemia duration. This finding highlights the potential benefit of early control and treatment of hyperuricemia for the prevention of DM.

Another interest implications of our study, particularly when viewed in the context of other studies, is that the time course of SUA accumulation was taken into account. The results showed individuals with the same cumulative exposure to SUA but with a greater fraction of that exposure occurring earlier in life had a greater risk of incident DM risk. That means the same cumSUA exposure accumulated earlier is associated with higher risk of DM compared with later in life. These data underscore the dependence of risk, not just on the present amount of cumSUA level, but also the SUA versus time history, and offer a model to quantify the modulation of risk by the time course of SUA. Future clinical trials of lowering SUA early should be conducted to investigate whether there is a major reduction in DM incidence compared to risk reduction started later.

There are several biological mechanisms underlying the relationship of cumSUA and its time course with DM. Several in vitro and in vivo studies showed that high SUA levels can induce oxidative stress, which has been established as a pathological pathway for the development of DM [36]. First, high cumSUA produces oxidative stress via pathways involving the activation of nicotinamide adenine dinucleotide phosphate oxidase and the generation of oxidized lipids and inflammatory mediators [6]. Second, high cumSUA can regulate enzymes associated with glucose and lipid metabolism primarily in the liver, adipose tissue, and skeletal muscle [37]. Through positive feedback, adipose tissue could produce and secrete additional SUA via xanthine oxidoreductase [38]. Third, elevated cumSUA would decrease insulin sensitivity and lead to insulin resistance through altering glucose metabolism [39]. High cumSUA can induce endothelial dysfunction and nitric oxide inhibition, which in turn contribute to insulin resistance and thus diabetes [40]. This is supported by findings that fructose-induced hyperuricemia in rats leads to insulin resistance along with other components of metabolic syndrome, and these conditions are improved by decreasing uric acid levels [6].

The strengths of our prospective study is that when assessing the association between cumSUA and DM, we considered the modulated effect of time course of cumSUA accumulation, which may provide additional information. However, our study still has several limitations. First, the use of urate-lowering agents was not available. Given the low rate of hyperuricemia therapy and the controversial benefit of pharmacological treatment for asymptomatic hyperuricemia, our conclusion would not meaningfully change. Second, due to the large sample, our cohort did not take oral glucose tolerance test and measure hemoglobin A1c to diagnose DM. Considering the same diagnosis criteria being used in all the participants which will lead to nondifferentiated misclassification, the association of cumSUA and its time course with DM would be underestimated. We therefore excluded participants diagnosed with diabetes less than 2 years to avoid misclassification at baseline and increase specificity of diabetes diagnosis. Finally, our population is from a region in North China; therefore, the finding may not be generalized.

## Conclusions

In conclusion, incident DM risk depends on both long-term cumulative exposure to SUA, and importantly, on the time course of SUA accumulation. The same cumulative exposure acquired earlier in life, compared with later, leading to a greater risk increase. The findings stress the importance of optimal SUA control starting early in life for preventing or reducing incident DM.

## Supplementary information

Supplemental Material

## Data Availability

Data are available to researchers on request for purposes of reproducing the results or replicating the procedure by directly contacting the corresponding author.

## Code availability

Code is available to researchers on request for purposes of reproducing the results or replicating the procedure by directly contacting the corresponding author.
