# Supplementary material for: Time course of serum uric acid accumulation and the risk of diabetes mellitus
Source: Nutr Diabetes. 2022 Jan 10;12:1. doi: 10.1038/s41387-021-00179-8 (PMC8748907; doi:10.1038/s41387-021-00179-8)
Supplement: Supplementary file 1 — Supplemental Material [file 41387_2021_179_MOESM1_ESM.docx]

**SUPPLEMENTAL MATERIALS**

Table S1. Baseline characteristics of included and excluded participants owing to missing data

| Characteristics | Excluded  (n=11222) | Included  (n=46434) | *P* value | ASD, % |
| --- | --- | --- | --- | --- |
| Age, years | 50.30±12.58 | 48.66±11.97 | <0.0001 | 13.31 |
| Male, n (%) | 8896 (79.27) | 35175 (75.75) | <0.0001 | 8.44 |
| High school or above, n (%) | 1884 (18.21) | 11049 (24.33) | <0.0001 | 14.99 |
| Income≥800RMB, n (%) | 1206 (11.66) | 7125 (15.70) | <0.0001 | 11.76 |
| Current smoker, n (%) | 3310 (30.80) | 15829 (34.88) | <0.0001 | 8.70 |
| Current alcohol, n (%) | 3529 (32.83) | 18126 (39.92) | <0.0001 | 14.79 |
| Active physical activity, n (%) | 9611 (93.08) | 41340 (91.23) | <0.0001 | 6.87 |
| Hypertension, n (%) | 991 (8.83) | 4652 (10.02) | 0.0001 | 4.07 |
| Dyslipidemia, n (%) | 411 (3.66) | 2526 (5.44) | <0.0001 | 8.54 |
| Antihypertensive agents, n (%) | 839 (7.48) | 4069 (8.76) | <0.0001 | 4.71 |
| Lipid-lowering agents, n (%) | 67 (0.60) | 367 (0.79) | 0.0335 | 2.33 |
| Body mass index, kg/m^2^ | 24.80±3.37 | 24.86±3.42 | 0.1110 | 1.70 |
| Fasting blood glucose, mmol/L | 5.01±0.65 | 5.00±0.66 | 0.4405 | 0.82 |
| Systolic blood pressure, mmHg | 129.26±20.33 | 127.42±19.63 | <0.0001 | 9.21 |
| Diastolic blood pressure, mmHg | 83.05±11.82 | 82.17±11.28 | <0.0001 | 7.56 |
| eGFR, mL/min/1.73m^2^ | 80.20±27.03 | 84.38±24.52 | <0.0001 | 16.20 |
| hs-CRP, mg/L | 2.20±5.57 | 2.25±6.19 | 0.4319 | 0.89 |

Abbreviations: ASD, absolute standardized difference; eGFR, estimated glomerular filtration rate; hs-CRP, high-sensitivity C-reactive protein.

Continuous variables were presented as means along with standard deviations. Categorical variables were presented as percentages. Given the large study population in our study, *P*<0.05 in the comparison indicates statistical significance but may not mean clinical significance. Therefore, baseline characteristics between excluded and included participants were compared with ASD, the indicator >10% is approximately equivalent to *P* value less than 0.05, indicating a significant imbalance.

Table S2. Baseline characteristics of participants according to quartiles of cumulative serum uric acid.

| Characteristics | Overall | Cumulative serum uric acid, μmol/L × year | | | | *P* value |
| --- | --- | --- | --- | --- | --- | --- |
|  |  | Q1 | Q2 | Q3 | Q4 |  |
| No. of participants | 46434 | 11608 | 11609 | 11609 | 11608 |  |
| Age, years | 48.66±11.97 | 46.53±10.97 | 48.33±11.74 | 50.06±12.02 | 49.74±12.77 | <0.0001 |
| Male, n (%) | 35175 (75.75) | 6510 (56.08) | 8281 (71.33) | 9581 (82.53) | 10803 (93.07) | <0.0001 |
| High school or above, n (%) | 11049 (24.33) | 2323 (20.41) | 2701 (23.77) | 2700 (23.89) | 3325 (29.23) | <0.0001 |
| Income≥800RMB, n (%) | 7125 (15.70) | 1239 (10.89) | 1644 (14.48) | 1858 (16.45) | 2384 (20.97) | <0.0001 |
| Current smoker, n (%) | 15829 (34.88) | 2509 (22.06) | 3461 (30.47) | 4472 (39.61) | 5387 (47.40) | <0.0001 |
| Current alcohol, n (%) | 18126 (39.92) | 2807 (24.66) | 3837 (33.79) | 4989 (44.17) | 6493 (57.11) | <0.0001 |
| Active physical activity, n (%) | 41340 (91.23) | 10355 (91.17) | 10277 (90.64) | 10266 (91.15) | 10442 (91.96) | 0.0056 |
| Hypertension, n (%) | 4652 (10.02) | 541 (4.66) | 829 (7.14) | 1278 (11.01) | 2004 (17.26) | <0.0001 |
| Dyslipidemia, n (%) | 2526 (5.44) | 290 (2.50) | 455 (3.92) | 664 (5.72) | 1117 (9.62) | <0.0001 |
| Antihypertensive agents, n (%) | 4069 (8.76) | 451 (3.88) | 706 (6.08) | 1111 (9.57) | 1801 (15.52) | <0.0001 |
| Lipid-lowering agents, n (%) | 367 (0.79) | 36 (0.31) | 70 (0.60) | 99 (0.85) | 162 (1.40) | <0.0001 |
| Body mass index, kg/m^2^ | 24.86±3.42 | 24.07±3.38 | 24.45±3.35 | 25.02±3.32 | 25.90±3.34 | <0.0001 |
| Fasting blood glucose, mmol/L | 5.00±0.66 | 5.00±0.66 | 5.01±0.66 | 4.99±0.65 | 5.02±0.64 | 0.0003 |
| Systolic blood pressure, mmHg | 127.42±19.63 | 124.5±18.95 | 126.22±18.8 | 128.14±19.65 | 130.82±20.52 | <0.0001 |
| Diastolic blood pressure, mmHg | 82.17±11.28 | 80.63±11.05 | 81.60±10.83 | 82.52±11.24 | 83.95±11.70 | <0.0001 |
| eGFR, mL/min/1.73m^2^ | 84.38±24.52 | 85.98±26.13 | 84.45±26.08 | 84.26±22.20 | 82.82±23.35 | <0.0001 |
| hs-CRP, mg/L | 2.25±6.19 | 1.76±3.97 | 2.01±7.23 | 2.50±6.72 | 2.74±6.29 | <0.0001 |

Continuous variables were presented as means along with standard deviations. Categorical variables were presented as percentages.

Abbreviations: eGFR, estimated glomerular filtration rate; hs-CRP, high-sensitivity C-reactive protein.

**Year 2006**

**Year 2008**

**Year 2010**

**CumSUA calculation**

**Follow-up for outcomes ascertainment**

**End of follow-up**

**(December 2017)**

**Figure S1. The time line of the study**

Abbreviations: cumSUA, cumulative serum uric acid


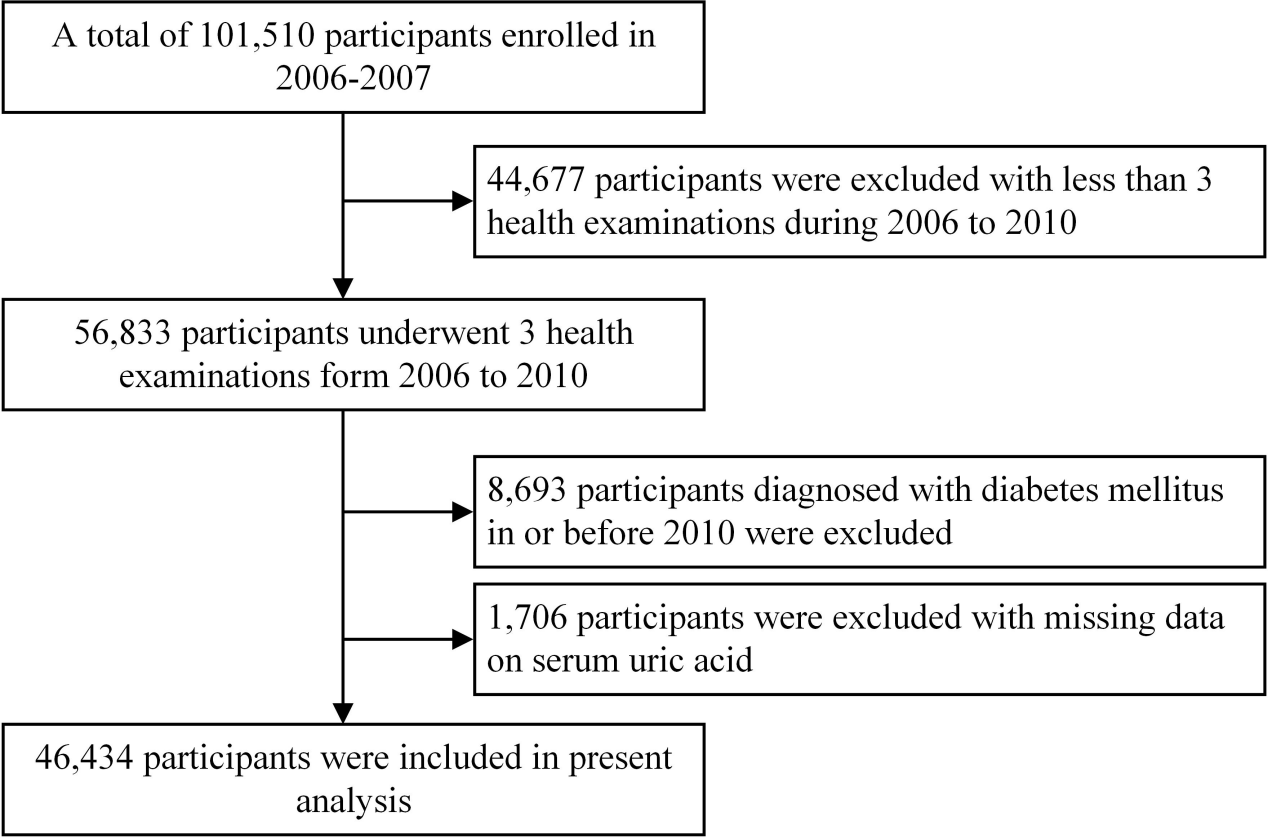


**Figure S2. The flowchart of the study**
